# Quality of life and people living with AIDS: relationship with sociodemographic and health aspects^1^

**DOI:** 10.1590/0104-1169.3350.2455

**Published:** 2014

**Authors:** Tadeu Lessa da Costa, Denize Cristina de Oliveira, Antonio Marcos Tosoli Gomes, Gláucia Alexandre Formozo

**Affiliations:** 2 PhD, Adjunct Professor, Universidade Federal do Rio do Janeiro - Campus Macaé, Macaé, RJ, Brazil; 3 PhD, Full Professor, Faculdade de Enfermagem, Universidade do Estado do Rio de Janeiro, Rio de Janeiro, RJ, Brazil

**Keywords:** Acquired Immunodeficiency Syndrome, Quality of Life, Nursing

## Abstract

**OBJECTIVE:**

to analyze the relationship of sociodemographic and health dimensions with the quality of life of people living with the human immunodeficiency virus.

**METHOD:**

descriptive and quantitative study. The subjects were 131 seropositive people treated in a specialized center of the Norte-Fluminense municipality, Brazil. A form with sociodemographic and health data was applied, as well as the World Health Organization instrument for the assessment of the quality of life of people with the human immunodeficiency virus.

**RESULTS:**

the statistical analysis revealed a significant difference in the assessment of the various dimensions of quality of life by the subjects for gender, education, employment, personal income, medical condition, self-perception of sickness, history of hospitalizations, and bodily alterations due to the antiretroviral drugs.

**CONCLUSION:**

professional nursing and health care, as well as public policies in the area, should valorize the quality of life approach, considering the conditions related to its configuration.

## Introduction

Quality of life (QoL) constitutes a field of progressive academic interest, given its potential. The development of the concept and its incorporation into the healthcare sector is basically due to: epidemiological studies on happiness and well-being; the search for new social indicators of health; the lack of objective measures of the results of biotechnologies; the positive psychology movement; valorization of the satisfaction of the client; and the need for humanization in health programming and care^(^ 1 ^)^. The QoL construct has also contributed to the comprehension of the factors involved in the existence of people infected by the Human Immunodeficiency Virus (HIV) and the disease that it causes, Acquired Immunodeficiency Syndrome (AIDS)^(^ 2 ^)^. This is due to the fact that, despite the increase in lifespan after HIV infection, due to the development of high activity antiretroviral therapy (HAART), many clinical and, in particular, psychosocial issues are still obstacles for the improvement of the QoL^(^ 3 ^-^ 4 ^)^. Thus, people living with HIV/AIDS (PLWHA) are still faced with significant difficulties such as: stigma and prejudice^(^ 5 ^)^; access to health care^(^ 6 ^)^; employment^(^ 7 ^)^; relationships with healthcare professionals^(^ 8 ^)^; experience of sexuality^(^ 9 ^)^; increased social support^(^ 9 ^-^ 10 ^)^; family relationships^(^ 4 ^,^ 10 ^)^; and bodily alterations and perception of self-image^(^ 4 ^)^. The World Health Organization (WHO), in turn, has invested in the approach to QoL, having constructed the Quality of Life Assessment Group (The WHOQoL Group) for this. This initiative culminated in the creation of a generic QoL assessment instrument called the WHOQoL-100, developed in a multicentric manner, with transcultural potential. Considering the particularities of living with AIDS, the WHOQoL-HIV was created, based on the above mentioned instrument^(^ 11 ^)^.

Considering the complexity in the conceptualization of QoL, the WHO defines it as "an individual's perception of their position in life in the context of culture and value systems in which they live and in relation to their goals, expectations, standards and concerns"^(^ 11 ^)^. It involves a comprehensive assessment of the perception of the subjects regarding a set of domains, in the case of the WHOQoL-HIV these being: physical; psychological; level of independence; social relationships; environment; and spirituality, religion and personal beliefs (SRPB)^(^ 11 ^)^. Therefore, given that socio-demographic and health characteristics may have an impact on the assessment of QoL^(^ 2 ^,^ 5 ^,^ 7 ^,^ 9 ^-^ 10 ^)^, this work aimed to: analyze the relationship of sociodemographic and health dimensions with the assessment of the QoL of PLWHA. Despite the existence of studies on the assessment of the QoL of PLWHA, there are few studies using the construct with approach facets specific to the WHOQoL-HIV. This is especially the case for medium-sized and small Brazilian municipalities, the spatial delimitation of this study. The analysis of the quality of life is essential for the healthcare policies and services directed toward PLWHA, since this indicator values the perception of people about their own life and health. This therefore serves as a basis for directing the programmatic and professional investments in health^(^ 1 ^)^. Accordingly, it contributes to the design of nursing care for this group, while indicating dimensions for collective and individual approaches by the nurse.

## Method

This is a descriptive study with a quantitative approach, with PLWHA as the subject. Data collection took place in Specialized Care Service (SCS) for HIV/AIDS in a medium-sized municipality of the northern region of Rio de Janeiro state. The criteria for the inclusion of subjects in the study were: to be attended in the SCS at the time of the interviews; age greater than or equal to 18 years; HIV seropositivity; and to have mental conditions that enable participation. There was approval of the project by the Research Ethics Committee of the Rio de Janeiro State University, under protocol No. 017.3.2011 with the provision of the Terms of Free Prior Informed Consent. The data collection period was between May and October 2011. The sample was non-probabilistic, and the selection of the subjects by convenience, complying with the inclusion criteria mentioned above. For the sample size calculation, the information that the SCS had 979 patients in attendance was used as a basis. A finite sample was calculated, from the pilot study with 45 subjects. With the prevalence obtained from the pilot study of 11%, the confidence level of 95% and error of 5%, a sample of 131 participants was defined.

Two specific instruments were used in the data collection, one designed for this study, containing sociodemographic and health data; and the second developed by the WHO for the assessment of the dimensions related the quality of life: The WHOQoL-HIV, in its abbreviated version (*Bref*). The WHOQoL-HIV-*Bref* instrument has 31 questions/facets distributed among one overall perception component and six QoL assessment domains, these being: physical; psychological; level of independence; social relationships; environment; and SRPB^(^ 12 ^)^. This instrument was translated and validated in Brazil, with acceptable reliability and consistency for the scale and its domains^(^ 13 ^)^. The questions of the WHOQoL HIV-*Bref* are structured in a Likert type scale with five grades depending on the nature of the domains and facets. Completion was carried out by the study subjects, unless this was impossible due to reading difficulties^(^ 13 ^)^.

The sociodemographic and health variables collected were: gender; age; education level; work; personal income; sexual orientation; affective-sexual partner; number of children; municipality of residence; number of people in the household; condom use; main source of information about AIDS, dichotomously categorized as healthcare professional/service and other means; time since diagnosis of HIV seropositivity; clinical condition (asymptomatic/symptomatic); self-perception of sickness; hospitalizations; HAART use; duration of HAART use; stage of HIV infection; and viral load. Regarding the data collection, it should be noted that this took place in a space assigned by the SCS in which the study took place, with the application of the instruments on all days of the week. The invitation for the participation of the subjects took second position to the medical consultation, with the substitution of those people who did not have an interest in joining the study.

Descriptive statistical analysis of the sociodemographic and health questionnaire data was performed with the aid of the Statistical Package for the Social Sciences (SPSS) 17.0. For the analysis of the data from the WHOQoL-HIV-*Bref*, initially, the WHO guidelines in its syntax were followed. The scores of the questions within each QoL domain are used to calculate the domain score, this being the mean of the scores of the questions. The mean domain scores were then multiplied by 4 to make the resulting scores comparable with those used in the WHOQoL-100, with the values varying between the minimum of 4 and maximum of 20^(^ 14 ^)^. For the statistical comparison between the sociodemographic data, health data and the QoL assessment, the normality test of the distributions of the variables of the six WHOQoL-HIV-*Bref* domains was first carried out. This was performed through the *Kolmogorov-Smirnov* test, using* SPSS 17.0*. Considering the results of this test, it was necessary to adopt the parametric statistical comparison tests Student's t-test (comparison between two subgroups); and Analysis of Variance (ANOVA) (comparison of three or more subgroups), for the domains with normal distributions: Physical; Psychological; Level of independence; Social Relationships; and Environment; and the non-parametric tests the Mann-Whitney test (comparison between two groups), and the Kruskall-Wallis test (comparison involving three or more groups) for the non-normal distribution of the SRPB domain. For the performance of Student's t-test and ANOVA, Levene's test was also used to examine the homogeneity of variances. In the case of heterogeneity of the variances, the non-parametric tests were employed. The multiple comparisons of the post-test means were carried out using Tukey's-Honest Significant Differences (HSD) test for the ANOVA and by the comparison between each pair of variable categories, through the Mann-Whitney test, in the case of the Kruskal-Wallis test. In all the comparisons, a significance level p<0.05 was adopted.

## Results

Table 1 presents the data related to the characterization of the subjects.

**Table 1:** Distribution of the people living with HIV/AIDS according to sociodemographic and health variables. Norte-Fluminense municipality, RJ, Brazil, 2011

Variable		n	%
Gender			
	Male	68	51.9
	Female	63	48.1
Age group			
	20-29	24	18.3
	30-39	38	29
	40-49	43	32.8
	50-59	19	14.5
	≥60	7	5.3
Schooling			
	Had not studied	2	1.5
	Incomplete elementary education	44	33.6
	Complete elementary education	36	27.5
	Complete high school education	39	29.8
	Complete higher education	10	7.6
Marital status			
	Single/no steady partner	54	41.2
	Married/living with partner/stable union	61	46.6
	Steady partner, however, not living together	16	12.2
Sexual orientation			
	Heterosexual	103	78.6
	Homosexual/bisexual	28	21.4
Exposure category			
	Sexual	127	96.9
	Other	4	3.1
Time since diagnosis (years)			
	≤1.9	25	19.1
	2 to 5	35	26.7
	5.1 to 10	38	29.0
	≥10.1	33	25.2
High activity antiretroviral therapy use			
	Yes	101	77.1
	No	30	22.9
Infection stage			
	Stage 1 (≥500 cd4/mm^3^)	56	42.7
	Stage 2 (499 to 200 cd4/mm^3^)	48	36.6
	Stage 3 (<200 cd4/mm^3^)	10	7.6
	Stage 4 (unknown value)	17	13.0
Employment			
	Yes	71	54.2
	No	60	45.8
Employment status			
	Self-employed	33	25.2
	Formally employed	32	24.4
	Unemployed	22	16.8
	Retired/pensioner	32	24.4
	Other	12	9.2
Individual income (minimum wages)			
	Without income	22	16.8
	≤1	39	29.8
	1.1 to 3	49	37.4
	3.1 to 5	10	7.6
	≥5	11	8.4
Total		131	100

Considering the comparative analysis between the assessment of the QoL and the sociodemographic and health data there was no statistically significant difference (p<0.05) in any of the domains for the following variables: sexual orientation; marital status; condom use; age group; municipality of residence; number of children; number of people in the household; source of information about AIDS; time since diagnosis; HAART use; duration of HAART use; infection stage; and viral load. In relation to the sociodemographic variables with statistically significant differences, there was a difference for the male and female genders in three domains: psychological; environment; and SRPB, all with higher scores among the men (Table 2). Concerning the level of education, a statistically significant difference was only identified in the level of independence domain. Tukey's post-hoc test showed that this effect occurred more specifically among people with incomplete elementary education and higher education levels. Nevertheless, it was noticed that the mean scores increased as the level of education increased (Table 2). It should be noted that the subgroup that reported not having studied was excluded from the comparison due to being represented by only two subjects.

**Table 2:** Distribution of the Quality of Life assessment domains, according to the sociodemographic variables. Norte- Fluminense municipality, RJ, Brazil, 2011

Variables		n	%	Physical	*p* value	Psychological	*p* value	Level of independence	*p* value
Gender					0.185		0.009^†^		0.367
	Male	68	51.91	14.20		15.00		14.26	
	Female	63	48.09	13.40		13.60		13.75	
Education					0.163		0.098		0.031^†^
	Had not studied	2	1.53	12.00		12.40		11.00	
	Elementary Incomp.	44	33.59	13.36		13.65		13.14*	
	Elementary Comp.	36	27.48	14.50		14.51		14.33	
	High school comp.	39	29.77	13.33		14.69		14.31	
	Higher comp.	10	7.63	15.50		16.16		16.00*	
Employment					0.011^†^		0.002^†^		0.000^†^
	Yes	71	54.19	14.55		15.21		14.97	
	No	60	45.80	12.93		13.37		12.85	
Personal Income					0.032^†^		0.128		0.016^†^
	Without income	22	16.79	12.23*		13.09		12.86	
	≤1 MW	39	29.77	13.10		14.05		13.10	
	1.1 to 3 MWs	49	37.40	14.73*		14.81		14.78	
	3.1 to 5 MWs	10	7.63	14.80		15.60		14.80	
	>5 MW	11	8.40	14.45		14.98		15.27	
Variables				Social relationships	* p* value	Environment	* p* value	SRPB^‡^	* p* value
Gender					0.289		0.046^†^		0.018^†^
	Male			14.79		13.10		14.75	
	Female			14.16		12.24		13.16	
Education					0.101		0.235		0.069
	Had not studied			11.50		11.50		11.50	
	Elementary Incomp.			13.98		12.68		14.25	
	Elementary Comp.			14.19		12.87		13.46	
	High school comp.			14.92		14.00		16.70	
	Higher comp.			16.70		12.70		14.02	
Employment					0.019^†^		0.033^†^		0.044^†^
	Yes			15.13		13.11		14.56	
	No			13.73		12.18		13.30	
Personal Income					0.200		0.030^†^		0.027^†^
	Without income			13.45		12.25		12.05*	
	≤1 MW			13.90		12.05*		14.03*	
	1.1 to 3 MWs			15.02		12.81		14.12*	
	3.1 to 5 MWs			15.30		13.50		15.50*	
	>5 MW			15.55		14.55*		15.73*	

* Statistically significant difference after multiple comparison of means †p

Regarding the work activity of the subjects, statistically significant differences were seen for all six domains of the WHOQoL-HIV-*Bref*. Accordingly, higher scores were identified among those who were, in any way, included in the labor market (Table 2). For the personal income variable, initially, it should be noted that the reference value of one minimum wage was, at the time of data collection, R$565.00. Statistically significant differences were identified in four domains: physical; level of independence; environment; and SRPB. There was an increase in the scores in the domains mentioned according to increases in the corresponding minimum wage band. However, Tukey's multiple comparison indicated statistically significant differences in the physical domain only for the subjects without income and those with incomes of 1.1 to 3 minimum wages (Table 2). For the level of independence domain, the test mentioned did not specify the specific difference between the subgroups, however, lower scores were noticed among individuals without income or with less than or equal to 1 minimum wage compared to the others with 1.1 wages or more (Table 2) .

For the environmental domain, Tukey's test revealed a statistically significant difference between the subgroup with 1 wage or less and those with more than 5 minimum wages. Accordingly, for the SRPB domain, the pairwise post-hoc procedure with the *Mann-Whitney* test characterized the individuals without income as having scores significantly lower than the others (Table 2). Regarding the clinical condition variable, as perceived by the respondents, statistically significant differences can be observed in all the domains of the WHOQoL-HIV-*Bref*, except for the SRPB domain. In all the domains with statistical differences, the asymptomatic subjects presented higher scores than the symptomatic subjects (Table 3). Considering the self-perception of sickness, statistically significant differences were observed in all six domains. However, the scores among those not self-evaluated as sick were higher in all cases (Table 3). With regards to hospitalizations for AIDS, a statistically significant difference was only perceived in the level of independence domain, with higher scores among the subjects who did not have the experience of hospitalization due to the infection (Table 3).

**Table 3:** Distribution of the Quality of Life assessment domains according to the sociodemographic variables. Norte- Fluminense municipality, RJ, Brazil, 2011

Variables		n	%	Physical	*p* value	Psychological	*p* value	Level of independence	*p* value
Clinical condition					0.000*		0.000*		0.000*
	Asymptomatic	91	69.47	15.05		15.16		14.98	
	Symptomatic	40	30.53	10.98		12.56		11.78	
Self-perception of sickness					0.000*		0.000*		0.000*
	Yes	35	26.72	10.26		12.25		11.09	
	No	96	73.28	15.10		15.14		15.06	
Hospitalization for AIDS					0.364		0.510		0.034*
	Yes	48	36.64	13.44		14.13		13.25	
	No	83	63.36	14.02		14.51		14.43	
Bodily alterations due to High activity antiretroviral therapy					0.017*		0.032*		0.073
	Yes	81	80.20	13.33		14.07		13.46	
	No	20	19.80	15.40		15.84		14.85	
Variables				Social relationships	*p* value	Environment	*p* value	SRPB^†^	*p* value
Clinical condition					0.007*		0.039*		0.114
	Asymptomatic			15.02		12.98		14.36	
	Symptomatic			13.28		12.01		13.13	
Self-perception of sickness					0.001*		0.001*		0.001*
	Yes			12.89		11.46		12.09	
	No			15.07		13.13		14.68	
Hospitalization for AIDS					0.814		0.662		0.950
	Yes			14.40		12.81		13.98	
	No			14.54		12.61		13.99	
Bodily alterations due to High activity antiretroviral therapy					0.409		0.158		0.732
	Yes			14.36		12.63		14.21	
	No			15.15		13.50		14.00	

* *p<0.05

† Domains spirituality, religion and personal beliefs

The variable covering physical-bodily alterations associated with HAART use presented a statistically significant difference in the physical and psychological domains, with higher scores among PLWHA who did not perceive these alterations. A p value close to 0.05 was also observed in the level of independence domain (Table 3).

## Discussion

Considering the comparison with other studies, different results from the present study were observed for the variables: Age group, with the highest scores among the youngest (20 to 39 years)^(^ 15 ^-^ 16 ^)^; time since diagnosis, with higher scores in the SRPB domain between 2 and 5 years^(^ 15 ^)^; and CD4+ count, with higher scores in the physical domain^(^ 17 ^)^ and in the level of independence^(^ 15 ^)^ for higher cell values. Regarding the influence of HAART use, there was a different result from previous studies, which may have arisen from aspects possibly related to adherence to the treatment, not analyzed in this study, or bodily changes due to the medications, identified by the majority of the subjects in this study. In other investigations in the area, lower QoL was found in the level of independence domain among the users of these medications^(^ 15 ^)^. In other studies, the negative influence of antiretroviral drugs was identified in the physical and social domains among PLWHA in the first year of use, with better assessments in the SRPB and environment domains in the second year^(^ 18 ^)^. An additional study found the use of HAART to be a predictor of better QoL. This was especially the case when there was permanence of the therapeutic regimen and few adverse reactions^(^ 19 ^)^. 

With regards to the influence of the viral load on QoL, the findings of this study were similar to others in the literature^(^ 15 ^)^, in which this variable yielded no statistically significant difference in any of the domains of analysis. However, given that lower CD4 counts and higher viral loads are associated with symptoms of the disease, it is possible that their expressions exist, even indirectly, in other facets of the QoL^(^ 15 ^)^. In the case of this study, this may occur with the clinical condition variable, with statistically significant differences in five of the six domains.

For the gender variable, the results were similar to other studies^(^ 13 ^,^ 15 ^-^ 17 ^)^, with higher scores in the QoL domains among men. In this sense, although some authors question whether there are indeed differences in the QoL perception between men and women or just a differences in the style of responses, it is known that the socioeconomic and cultural reality in general proves unfavorable for females^(^ 13 ^)^. Thus, a different perspective would be necessary for women living with HIV/AIDS, considering their vulnerabilities, which refer to the important differences in the cultural, social and economic aspects that generate unequal opportunities in the protection, promotion and maintenance of health^(^ 16 ^)^.

The results concerning the influence of the education level on QoL presented a similar pattern to other studies^(^ 17 ^,^ 20 ^)^, with higher scores in the perception of the subjects for the higher strata of education. This aspect highlights the impact of social factors, such as the educational level, on QoL. Thus, it can be understood that the educational level could influence the self-management skills faced with the disease and its various demands^(^ 13 ^)^. It is thought that there should be easier access to and comprehension of the information on the issues relevant to the disease.

Regarding the inclusion of PLWHA in the working world, the findings of this study agree with and broaden the perspectives of other studies^(^ 16 ^,^ 20 ^)^, as statistically significant differences were found in all six QoL domains. It is comprehended that physical and mental health can benefit from working activity in an interactive and mutually reinforcing process^(^ 7 ^)^. This is because work can be a source of suffering and stress, however, also a source of pleasure, self-realization and identity formation^(^ 21 ^)^. In the case of PLWHA, in addition to access to the material conditions of existence, work allows a deviation from thinking about the often negative issues of the disease, creating feelings of usefulness and productivity. It can also have the potential of broadening social contacts, although commonly some barriers are observed related to the employability of these subjects^(^ 21 ^)^. The low level of education, which was prevalent among the study participants, may be an additional factor limiting access to work and better positions in this field, indicating the importance of specific policies. Although the design of this study did not allow for a conclusive statement, a possible influence of gender on this dimension could also occur, given the pattern of lower assessments of QoL observed among the women.

Considering personal income, the results are similar to studies carried out in Brazil, with the difference that they found no significant difference in the SRPB domain, while a difference was noted in the social relationships domain^(^ 16 ^-^ 17 ^)^. As occurred in the work variable, these findings also highlight the impact of the socioeconomic levels on the QoL of these subjects, included together with education because they are indicators involved in the characterization and implications of the tendency towards a poverty related epidemic in the country^(^ 22 ^)^.

With regards to the clinical condition of PLWHA, there are reports in the literature of differences in the QoL assessment between asymptomatic and symptomatic people, with higher scores for the first, especially in the physical domain^(^ 17 ^)^. In the present study, however, as in another study^(^ 13 ^)^, there was no influence of this variable in any other domains apart from the physical dimension. This result reinforces the perspective of a potential relationship between the biological aspects and those of a psychosocial nature. The clinical condition perceived as symptomatic, caused more negative assessments in the physical and level of independence domains, as well as in the psychological and social relationships domains.

Regarding the variable self-perception of sickness, it was initially thought that in the data set of the study it could have redundancy with the clinical condition. However, it is believed that this does not occur, at least in a complete way, as the SRPB domain was not involved in the clinical condition. Thus, this can strengthen studies that show the incorporation of subjectivity in the QoL assessment, in view of its contributions in the context of actions and interventions in health^(^ 1 ^)^.

The results concerning the influence of hospitalizations due to the disease on the level of independence QoL domain may be related to the fact that hospitalization occurred for the treatment of any clinical condition, which probably generated sequel and/or began to interfere, even temporarily, in the activities of daily life. It may also have lead to the need to take more medications or a reduction in the perception of physical conditions compatible with work^(^ 23 ^)^. Furthermore, the majority of the study subjects were on HAART and many had a CD4 count equal to or greater than 500CD4/mm^3^, reducing the chances of experiencing hospitalization and its negative consequences.

In relation to the findings concerning the influence of bodily alterations due to HAART use on the physical and psychological QoL domains, this is thought to have occurred due to adverse reactions caused by the medication and by the dependence on continuous treatment in the quotidian. Thus, this reinforces the recommendations on the importance of valuing these aspects in the monitoring of HIV seropositive clients, taking into account the impact of these aspects on the QoL, treatment adherence and health status^(^ 23 ^)^.

Considering the developments regarding the programmatic facets inherent to the set of findings on the QoL of PLWHA, it is reiterated that an important component for controlling the epidemic in the country is related to ensuring the provision of specialized healthcare for this group. However, access to the health programs, services and interventions faced with the disease still occurs unevenly, resulting in the configuration of different vulnerability profiles^(^ 6 ^)^. This scenario, in turn, can be reduced by taking into consideration the guiding principles for public policy and professional actions in nursing and health based on the operating logic of QoL, which, in this study, indicates social determinants in combination with those of health already investigated in this study.

It is thought that nursing has an important role in evaluating and promoting the improvement of the QoL of PLWHA. This is because it has the person, health and environment among its metaparadigms, in convergence with the theoretical concepts around the multidimensional phenomenon of QoL.

## Conclusions

This study revealed the impact of the following conditions on the various QoL domains measured: gender; education; work; personal income; clinical condition; self-perception of sickness; hospitalizations due to AIDS; and bodily changes associated with HAART use. Among the aspects with implications for the QoL, the role played by inclusion in the labor market and the self-perception of PLWHA about whether they were sick or not should be particularly highlighted. This consideration is due to the fact that these conditions significantly impacted on all six QoL assessment domains adjusted to this group by the WHO. This indicates the relevance of these issues in individual or collective therapeutic approaches faced with living with the disease, given the challenges for seropositive individuals related to the world of work, as well as the need to promote active listening regarding the view of the clients about their own health.

The substantial increase in the lifespan of people living with the disease, currently observed, highlights the need to include other aspects, in addition to those eminently biomedical, regarding the relationship of the subjects with HIV/AIDS in their quotidian. It is therefore important to assume theoretical and attitudinal perspectives widely including the many facets that configure life with the disease, with the QoL being a construct with important potential contributions for the practice of nursing and health care for this group, as well as for the design of public policies in the area. The limitations to the study included: the use of a Likert type scale, self-reported by the subjects; and the inclusion of participants using HAART or not, taking into account the possible consequences of this in their lives.
